# Multiple Activities of *Punica granatum* Linne against Acne Vulgaris

**DOI:** 10.3390/ijms18010141

**Published:** 2017-01-12

**Authors:** Chia-Jung Lee, Lih-Geeng Chen, Wen-Li Liang, Ching-Chiung Wang

**Affiliations:** 1 PhD Program for Clinical Drug Discovery of Chinese Herbal Medicine, College of Pharmacy, Taipei Medical University, Taipei 11031, Taiwan; cjlee@tmu.edu.tw; 2 Department of Microbiology, Immunology and Biopharmaceuticals, College of Life Sciences, National Chiayi University, Chiayi 60004, Taiwan; lgchen@mail.ncyu.edu.tw; 3 School of Pharmacy, College of Pharmacy, Taipei Medical University, Taipei 11031, Taiwan; wenlee@tmu.edu.tw; 4 Orthopedics Research Center, Taipei Medical University Hospital, Taipei 11031, Taiwan

**Keywords:** acne, *Punicagranatum*, anti-inflammation

## Abstract

Acne is a common skin condition with sebum overproduction, hyperkeratosis, *Propionibacterium acnes* (*P*. *acnes*) and *Staphylococcus aureus*, and inflammation. *Punica granatum* (pomegranate) is well-known for its anti-inflammatory effects; however, few studies have discussed the anti-acne effects of pomegranate. In this study, we found that pomegranate extract (PG-E) significantly reduced *P*. *acnes*-induced edema in Wistar rat ears. Therefore, an evaluation platform using multiple pathogenic mechanisms of acne was established to explore the anti-acne effects of pomegranate. Results showed that PG-E inhibited bacterial growth and lipase activity. Through a bioguided-fractionation-isolation system, four hydrolysable tannins, punicalagin (**1**), punicalin (**2**), strictinin A (**3**), and granatin B (**4**), were isolated. Compounds **1** and **2** had greater anti-bacterial activities and anti-testosterone-induced HaCaT proliferative effects than the others. Compounds **1**, **3**, and **4** displayed lipase inhibitory effects. Compound **4** decreased cyclooxygenase-2 expression and downregulated prostaglandin E_2_ production in heat-killed *P*. *acnes*-treated RAW 246.7 cells. In conclusion, PG-E is abundant in hydrolysable tannins that display multiple anti-acne capacities, including anti-bacterial, anti-lipase, anti-keratinocyte proliferation, and anti-inflammatory actions. Hence, PG-E has great potential in the application of anti-acne and skin-care products, and punicalagin (**1**), the most effective component in PG-E, can be employed as a quality control marker.

## 1. Introduction

Acne vulgaris (acne), a common skin disease, usually appears in young adolescents with a hormone imbalance [1,2]. More than 85% of teenagers are affected by acne, and most of them continue to be affected into adulthood. In the United States, acne therapies cost approximately $1 billion per year, while more than $100 million is spent on over-the-counter acne products [3,4]. Major causes of acne formation are fourfold: (1) increasing sebum production by overactive oil glands; (2) retention hyperkeratosis, which blocks skin pores; (3) activities of normal skin bacteria (*Propionibacterium acnes* and *Staphylococcus aureus*); and (4) skin inflammation [5].

In sebum production, human sebum is composed of fatty acids, triglycerides, wax esters, and so on. Skin areas rich in sebaceous glands are positively correlated with acne lesions [2]. Nowadays, one therapeutic strategy for the increased sebum production is topical use of azelaic acid, a dicarboxylic acid found in rye, wheat, and barley, which can block the synthesis of fatty acids to avoid the overproduction of sebum. However, long-term use of azelaic acid results in skin depigmentation [6]. Evidences have shown that *Chamaecyparis obtuse* (*C*. *obtuse*) extract and tea tree oil were usually applied for the reduction of sebum productions. However, the therapeutic effect of *C*. *obtuse* is suboptimal and toxicity issue should be concerned in the use of tea tree oils [7]. In hyperkeratosis, androgens, such as testosterone, cause hyperresponsiveness that stimulates sebocytes and follicular keratinocytes, resulting in hyperplasia of the sebaceous glands [1]. *Artemisia capillaries* (*A*. *capillaries*) is reported for its anti-inflammatory effects against hepatic injury. Topical application of *A*. *capillaries* reduced hyperkeratosis of the epidermis, also suggesting the potential therapeutic activity to treat atopic dermatitis [8]. Retinoic acid and salicylic acid are typically use to treat hyperkeratinization, but these chemical agents also lead to skin irritation and desquamation [9]. In skin bacterial infections, oral anti-biotics such as tetracycline, erythromycin, and clindamycin are the most popular drugs for treating bacteria-induced acne at present. Overuse of antibiotics is a huge problem for modern medicine, because it results in anti-biotic resistance. Moreover, many limitations of anti-biotics were found when used as acne treatment. For example, tetracycline should be used in a fasting status, and one should avoid taking it with milk. Pregnant women and children are also discouraged from using tetracycline [10]. Several botanical extracts displayed the anti-skin bacterial effects, such as *Coriandrum sativum* leaf extract, but none of them discussed the multiple therapeutic effects against acne [11]. In anti-inflammation, non-steroid anti-inflammatory drugs (NSAIDs), such as ibuprofen, diclofenac, and ponstan, are typically used to inhibit biosynthesis of prostaglandin, which is an important mediator in inflammatory reactions. NSAIDs can rapidly decrease the inflammation of acne skin, but the commonest side effects of NSAIDs are peptic ulcers and liver toxicity [12]. In accordance with previous research, we examined the anti-acne effects of pomegranate extract (PG-E) in vivo and used multiple pathogenic mechanisms of acne vulgaris to establish an anti-acne system model. First, we used HaCaT cells, an immortal human keratinocyte cell line, as a target to simulate testosterone-induced epithelial cells and keratin accumulation. Increased bacterial lipase activity facilitates the synthesis of fatty acids and bacterial growth. In addition, *Propionibacterium acnes* (*P*. *acnes*) and *Staphylococcus aureus* (*S*. *aureus*) are the major pathogenic and most abundant bacteria on the skin surface of acne-affected skin. Eventually, the combination of bacteria with the above-described situations will result in inflammation of the skin. Hence, we used THP-1, a human monocytic cell line, as the target to simulate anti-inflammatory activities against different inducers. The pathogenic mechanism of acne and experimental design of our platform is summarized in Table 1. 

*Punica granatum* Linne (pomegranate) belongs to the Punicaceae family. In the Mediterranean area, pomegranate is commonly used as a fruit for therapeutic agents, foods, and cosmetics. However, in traditional Chinese medicine, pomegranate was recorded as an astringent agent [13]. Evidence has shown that the pharmacological effects of pomegranate are its anti-inflammatory, anti-oxidative, anti-lipoperoxidative, anti-bacterial, and anti-tumor activities [13,14,15,16,17]. In our previous study, four hydrolysable tannins, punicalagin (**1**), punicalin (**2**), strictinin A (**3**), and granatin B (**4**), were isolated from pomegranate using column chromatography combined with in vitro anti-inflammatory-guided fractionation [13]. These four compounds and the 70% acetone pomegranate extract displayed both in vitro and in vivo anti-inflammatory effects. However, few researchers have discussed the anti-acne effects of pomegranate. Therefore, an evaluation platform was used to explore the anti-acne effects of pomegranate. In addition, the principal anti-acne components of pomegranate are also discussed in this study. 

## 2. Results and Discussion

### 2.1. Pomegranate Attenuate P. acnes-Induced Wistar Ear Edema

**Figure 1:**
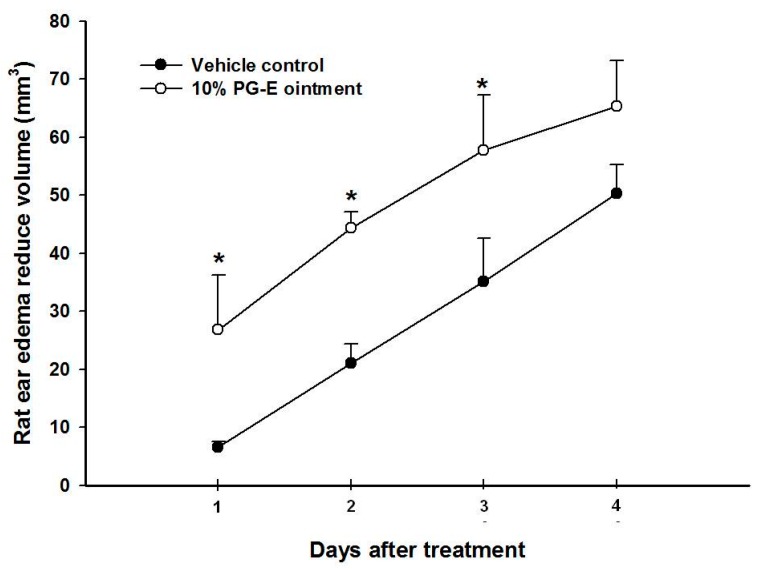
Anti-ear edema effects of pomegranate extract (PG-E) ointment against a *P*. *acnes* injection; * *p* < 0.05, compared to the vehicle group on the same day; Data are presented as the mean ± SD; Significance was calculated using Student’s *t*-test by SPSS software v.15; Each group contained eight rats.

In vivo anti-acne effects are difficult to assess because of a lack of animal models. We set up an animal model to simulate acne formation by directly injecting *P*. *acnes* into a rat’s ear and evaluated the in vivo anti-acne effects of PG-E. Wistar rat ears exhibit edema when live *P*. *acnes* is injected. As shown in Figure 1, rat ear edema in the PG-E ointment group was significant lower than that in the vehicle control group. In this model, live *P*. *acnes* was injected into Wistar rat ears. We first suggest that PG-E can inhibit the growth of *P*. *acnes*, resulting in a lower inflammatory status and less ear edema.

### 2.2. Pomegranate Displayed No Skin Irritation

As shown in previous studies, a modern therapeutic strategy for acne is to use retinoic acid or salicylic acid. However, skin irritation and desquamation are obvious side-effects of these chemical agents. In this study, a single-dose skin irritation test was performed by modifying the Draize test [18]. PG-E displayed no skin irritation in Wistar skin at 0.1–10 mg/site for 24–48 h, indicating the excellent safety of PG-E (data not shown). 

### 2.3. Pomegranate Significantly Inhibited P. acnes and S. aureus Growth

Because PG-E significantly attenuated *P*. *acnes*-induced Wistar ear edema, we continued to explore the in vitro activities and try to discover the mechanisms. PG-E displayed stronger anti-bacterial activity against *P*. *acnes* than against *S*. *aureus*. The diameter of the inhibition zone of PG-E against *P*. *acnes* ranged 11.3–17.1 mm. Moreover, the inhibition zone of PG-E against *S*. *aureus* ranged 12.6–15.9 mm. We calculated the ratio of the inhibition zone compared to penicillin. Ratios of the inhibition zone of PG-E against *S*. *aureus* and *P*. *acnes* were 0.451 and 0.544, respectively (Table 2).

Both the MIC (minimum inhibitory concentration) and MBC (minimum bactericidal concentration) of PG-E against *P*. *acnes* and *S*. *aureus* were 62.5 µg/mL. Several researchers showed that pomegranate peel extract displayed significant anti-bacterial effects against *Lactobacillus acidophilus*, *Staphylococcus epidermidis*, *Escherichia coli*, *Salmonella enteritidis*, and *Listeria monocytogenes* [19,20,21]. However, few researchers discussed the anti-bacterial effects of pomegranate against the skin-related bacterium, *P*. *acnes*. This is the first study to explore the anti-*P*. *acnes* effects and try to identify the active components of pomegranate. Through a bioguided-fractionation-isolation system, anti-microbial activities of four hydrolysable tannins in PG-E, punicalagin (**1**), punicalin (**2**), strictinin A (**3**), and granatin B (**4**), were tested. Results showed that Compounds **1** and **2** have significant anti-bacterial abilities against *P*. *acnes* and *S*. *aureus*, while the MIC of **1** against *P*. *acnes* and *S*. *aureus* were 6.25 µg/mL (1.3 µM) and 12.5 µg/mL (2.2 µM); MIC of **2** against *P*. *acnes* and *S*. *aureus* were 6.25 µg/mL (1.3 µM) and 12.5 µg/mL (2.2 µM), respectively. Compounds **3** and **4** were less effective against *P*. *acnes* and *S*. *aureus* (Table 3). In Reddy et al.’s study [15], Compounds **1** and **2** were also isolated from pomegranate juice. Compound **1** displayed significant anti-bacterial effects against *Pseudomonas aeruginosa*, *Candida albicans*, and *Cryptococcus neoformans*. Anti-oxidative effects were also found for **1** and **2**, suggesting that PG-E can act to augment human’s anti-bacterial and anti-oxidative capacities [15]. Anti-acne bacteria effects of tannin compounds in herbal extracts are also well-reported, i.e., tannin components from *Cassia tora*, *Garcinia mangostana*, *Momordica charantia*, *Phyllanthus emblica*, and *Terminalia arjuna* [22]. However, hydrolysable tannins in pomegranate are firstly reported for their multiple activities against acne in this study. 

### 2.4. Pomegranate Polyphenols Caused Shrinkage and Damage in P. acnes and S. aureus

**Figure 2:**
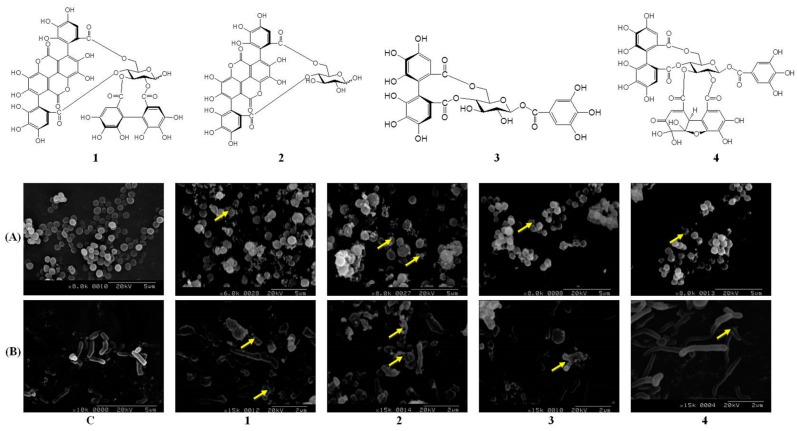
Shrinkage and damage of *S*. *aureus* (**A**) and *P*. *acnes* (**B**) after treatment with four hydrolysable tannins isolated from pomegranate extract (PG-E): punicalagin (**1**), punicalin (**2**), strictinin A (**3**), and granatin B (**4**); All photos were taken with scanning electron microscopy; C: control group; The concentration of **1** to **4** was 100 µg/mL; Data are from three separate experiments, the picture of one of which is shown; The arrow indicates a disruption site of bacteria; Scale bar of control group is 5 µm; Scale bar of four hydrolysable tannins against *S*. *aureus* and *P*. *acnes* is 5 µm and 2 µm, respectively.

Morphological changes are a clear indicator for monitoring the process of bacterial death. SEM, a powerful electron microscope, can be used to investigate diversification of a bacterium’s shape. Differences between treatments with four hydrolysable tannins in *P*. *acnes* and *S*. *aureus* are illustrated in Figure 2. As shown in Figure 2A, outer membranes of the control group were smooth and rod-shaped. However, after treatment with **1** or **2** (100 µg/mL) for 12 h, *S*. *aureus* produced around grains and exhibited bulging or deformation of its spherical shape. Intra-inclusions were found to have effluxed outside (representatives are indicated by arrows), and the bacteria were obviously swollen. In Rabie et al.’s study, the bacterial structure is an important point for its normal physiology, such as the outer membrane integrity [23]. We suggested that hydrolysable tannins in pomegranate damaged the structural integrity and bacterial proteins, leading to changes in the integrity and function of bacteria. Compounds **1** and **2** also significantly inhibited the growth of *P*. *acnes* (Figure 2B). However, *P*. *acnes* originally had a spherical shape and displayed a smooth, complete surface. The bacterial surface exhibited obvious shrinkage, damage to the surroundings, and increased granules after treatment with **1** and **2** for 12 h. Biofilms of bacteria play an important role in their virulence and pathogenicity. Another anti-bacterial strategy is to inhibit biofilm formation. It was reported that pomegranate significantly decreases biofilm formation by *S*. *aureus* [24]. Anti-biofilm formation capacities of pomegranate will be an important target in future studies.

### 2.5. Pomegranate Polyphenols Inhibited Lipase Activity

**Figure 3:**
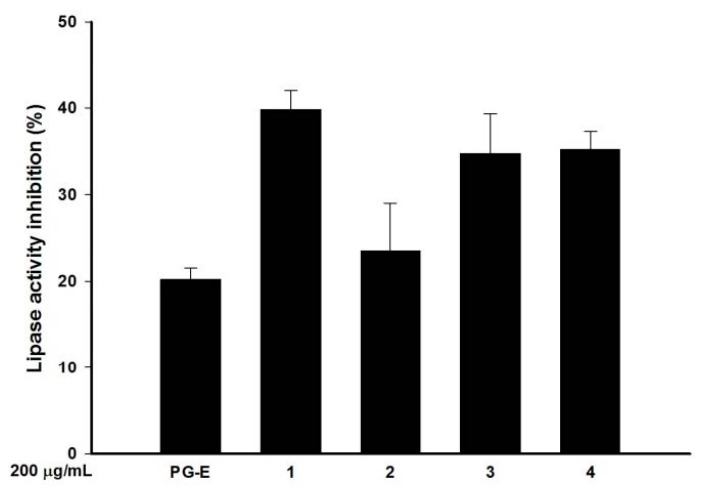
Lipase activity inhibition of pomegranate extract (PG-E) and four hydrolysable tannins included punicalagin (**1**), punicalin (**2**), strictinin A (**3**), and granatin B (**4**); The concentration of PG-E, and Compounds **1**–**4** was 200 µg/mL; Data are from three repeats.

Lipase is an enzyme that hydrolyzes lipids. Some bacteria, such as *P*. *acnes*, can secrete lipase to resolve lipids and facilitate lipid-related nutrient absorption from an external medium. In this study, a fluorometric assay of lipase activity was administrated with and without PG-E and the four hydrolysable tannins. Compared to the control, PG-E (200 µg/mL) inhibited lipase activity by 20% as shown by a decrease in lipase substrate degradation. The anti-lipase effects of the essential oil compositions of pomegranate peel extracts have been previously reported, but the principle active principle components were not discussed [25]. In this study, three hydrolysable tannins, 1, 3, and 4, at 200 µg/mL significantly inhibited the lipase activity by 39.8%, 34.7%, and 35.2%, respectively (Figure 3).

### 2.6. Pomegranate Polyphenols Inhibited HaCaT Cell Proliferation

**Figure 4:**
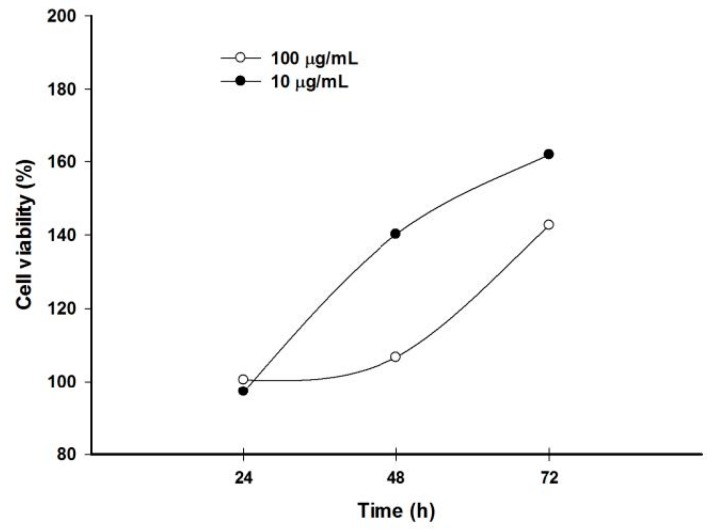
Testosterone significantly induced keratinocyte over-proliferation in a dose- and time-dependent manner.

Because keratinocyte over-proliferation is a crucial event in acne formation, testosterone was used as an inducer to stimulate HaCaT cell proliferation. Results showed that testosterone time- and dose-dependently induced HaCaT cell proliferation. Further, testosterone significantly induced proliferation of keratinocytes at 10 and 100 µg/mL for 72 h (Figure 4). 

**Figure 5:**
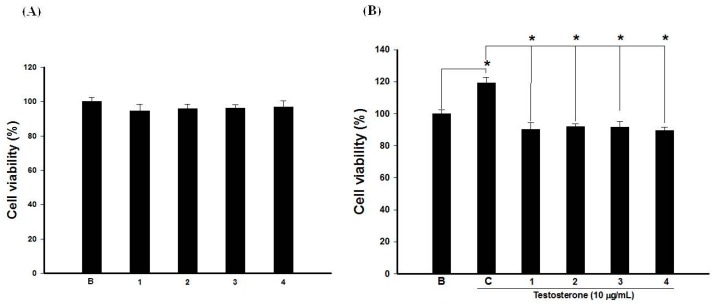
Cytotoxicity of different hydrolysable tannins (**A**); Anti-keratinocyte over-proliferative effects of hydrolysable tannins (**B**); B: blank; C: control; * *p* < 0.05, compared to the testosterone-only group; Data are presented as the mean ± SD; Significance was calculated using a one-way analysis of variance (ANOVA) via SPSS software v.15; Data are from three repeats.

Therefore, treatment with testosterone at 10 µg/mL for 72 h was used to assess which hydrolysable tannins could inhibit HaCaT cell proliferation. Result showed that four hydrolysable tannins could significantly decrease the testosterone-induced HaCaT cell proliferation at 50 µg/mL (Figure 5). Two research teams discussed the protective effects of pomegranate against UV-induced HaCaT oxidative stress and markers of photoaging [26,27]. Taken together with results of this study, we suggest that pomegranate can act as a UV protector and growth regulator of skin keratinocytes.

### 2.7. Pomegranate Polyphenols Attenuated Heat-Killed P. acnes-Induced NO and PGE_2_ Production by RAW 264.7 Cells

**Figure 6:**
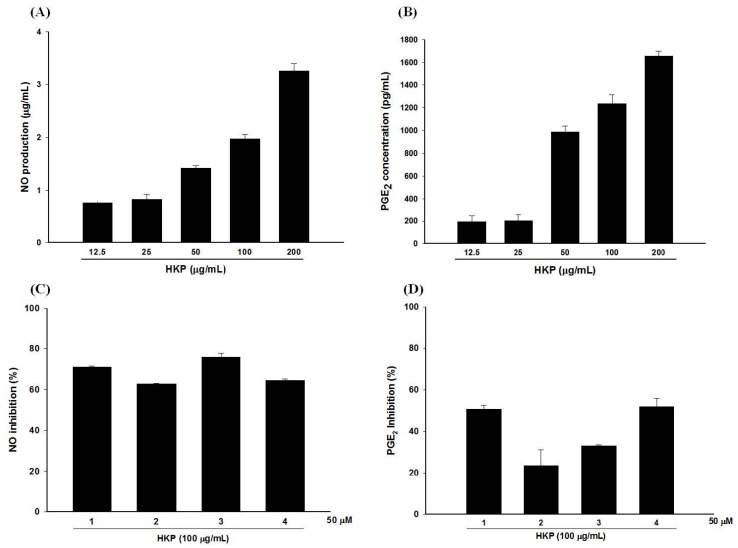
Heat-killed *P*. *acnes* (HKP) dose-dependently increased nitric oxide (NO) (**A**) and prostaglandin E_2_ (PGE_2_) (**B**) production by RAW 264.7 cells; Anti-inflammatory effects of hydrolysable tannins against HKP-induced NO (**C**) and PGE_2_ (**D**) production by RAW 264.7 cells; Data are from three repeats.

In our previous study, we showed the anti-inflammatory effects of PG-E and four hydrolysable tannins against LPS (lipopolysaccharide)-treated RAW 264.7 cells [12]. Moreover, in this study, we used HKP (heat-killed *P*. *acnes*), instead of LPS, to simulate the irritation and inflammation caused by a *P*. *acnes* infection. As shown in Figure 6A,B, HKP at 25–200 µg/mL dose-dependently and significantly induced NO (nitric oxide) and PGE_2_ (Prostaglandin E_2_) production by RAW 264.7 cells. However, a good relationship between the inflammatory response and the dose of HKP occurred at 50–200 µg/mL. Ultimately, we chose 100 µg/mL of HKP as our inflammation induction dosage. Compounds **1**, **2**, **3**, and **4** at 50 µM displayed over 50% NO inhibitory effects against HKP-induced RAW 264.7 cells (Figure 6C). In addition, **1** and **4** at 50 µM also significantly inhibited about 50% of PGE_2_ production in HKP-treated RAW 264.7 cells (Figure 6D).

### 2.8. Pomegranate Polyphenols Attenuated Heat-Killed P. acnes-Induced IL-8 and TNF-α Production by THP-1 Cells

**Figure 7:**
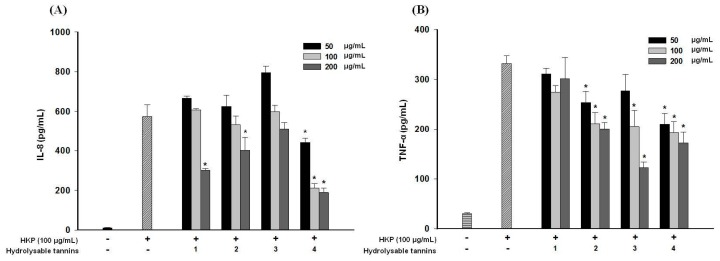
Anti-interleukin (IL)-8 (**A**) and tumor necrosis factor (TNF)-α (**B**) release from THP-1 cells after heat-killed *P*. *acnes* (HKP) induction; * *p* < 0.05, compared to the HKP-only group; Data are presented as the mean ± SD; Significance was calculated using a one-way analysis of variance (ANOVA) by SPSS software v.15; Data are from three repeats.

Cytokine, such as IL-8 (interleukin 8) and TNF-α (tumor necrosis factor-α), releases are also key points in the inflammatory status of human acne lesions [28]. As shown in Figure 7, HKP (100 µg/mL) obviously induced IL-8 and TNF-α production in a human monocytic cell line. In Figure 7A, the four hydrolysable tannins inhibited IL-8 and TNF-α production in dose-dependent manners at 50–200 µg/mL, and compound **4** had the strongest inhibitory activity. Anti-TNF-α activities of pomegranate polyphenols are shown in Figure 7B. Compounds **2**, **3**, and **4** exhibited significant inhibitory activities against TNF-α production, with **4** also displaying the strongest effect. This is the first time that Compound **4** has been reported to have anti-inflammatory cytokine effects. 

## 3. Materials and Methods 

### 3.1. General

Four active components of pomegranate, punicalagin (**1**), punicalin (**2**), strictinin A (**3**), and granatin B (**4**), were isolated and identified in our previous study (Figure 3) [13]. 3-(4.5-Dimethylthiazol-2-yl)-2.5-diphenyltetrazolium bromide (MTT), dimethyl sulfoxide (DMSO), lipopolysaccharide (LPS), and other chemicals were purchased from Sigma Industries (Saint Louis, MO, USA). Dulbecco’s modified Eagle medium (DMEM), Roswell Park Memorial Institute (RPMI) medium, fetal bovine serum (FBS), anti-biotics, and glutamine were purchased from GIBCO BRL (Grand Island, NY, USA). The bacterial culture equipment, including an anaerobic atmosphere by MGC AnaeroPack-Anaero and MGC AnaeroPack-Jar, were purchased from Mitsubishi Gas Chemical Company (Tokyo, Japan). Blood agar plates (BAPs), Bacto™ tryptic soy broth (TSB), and Bacto™ tryptic soy agar (TSA) were purchased from Difco (Detroit, MI, USA). The assay kits for tumor necrosis factor (TNF)-α, interleukin (IL)-1β, and IL-8 were purchased from eBioscience (San Diego, CA, USA). 

### 3.2. Preparation of Pomegranate Extract (PG-E)

Dried peels of pomegranate were purchased from a traditional Chinese medicine store in Taipei and identified by Hsien-Chang Chang. A voucher specimen (PG-01) was deposited in the School of Pharmacy, College of Pharmacy, Taipei Medical University (Taipei, Taiwan). Dried peels were extracted by homogenization with 70% acetone. The extracted solution was filtered, evaporated on a rotary evaporator, lyophilized, and called PG-E. PG-E and its active components were dissolved in 10% DMSO to a concentration of 10 mg/mL for subsequent experiments, stored at 4 °C, and used within 1 month. Serial dilutions of test solutions with culture medium were prepared before the in vitro assays. 

### 3.3. P. acnes-Induced Ear Edema in Wistar Rats

**Figure 8:**
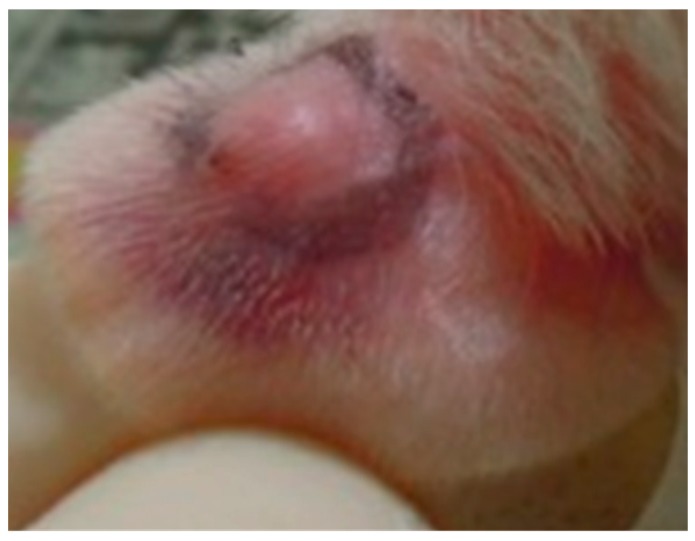
*P*. *acnes*-induced ear edema in Wistar rats.

The animal use protocol was reviewed and approved by the institutional Animal Care and Use Committee or Panel (IACUC/IACUP), Taipei Medical University (IACUC Approval no. LAC-97-0122). All methods involved in animal experiments were performed in accordance with the previous relevant guidelines and regulations. Female Wistar rats weighing 250 ± 20 g were bought from BioLASCO Taiwan (Yilan, Taiwan) and kept on a 12 h light/12 h dark cycle at 21 ± 2 °C with food and water ad libitum. *P*. *acnes* (8 × 10^7^ colony-forming units (CFU)/20 µL) was intradermally injected into the left ear of rats to induce edema (Figure 8). PBS (phosphate-buffered saline) was injected into the right ear as a vehicle control [29]. One hour before the injection, PG-E ointment (10% PG-E in hydrophilic ointment) was administered (eight rats per group) on the skin surfaces of the left ear. The perimeter of the ear edema included diameter and height after *P*. *acnes* or PBS injection after 1–4 days were measured by calipers. Volume of ear edema could be calculated using multiplication by diameter, height, and circular constant. In the vehicle control group, the rat ear edema reduce volume was calculated as the original volume of ear edema after the PBS injection minus the volume of ear edema after injection on days 1, 2, 3, and 4. In the PG-E ointment group, the rat ear edema reduce volume was calculated as the original volume of ear edema after the *P*. *acnes* injection minus the volume of ear edema administrated with PG-E ointment after injection on days 1, 2, 3, and 4. 

### 3.4. Skin Irritation

The skin toxicity of PG-E was evaluated from a single topical application in Wistar rats. Twenty-four hours before the test, the backs of rats were shaved free of hair (2.5 × 2.5 cm^2^ at each site) and checked for any abnormalities (integrity and allergies). An appropriate concentration of PG-E (50 µL per sample) was smeared onto the shaved back of a Wistar rat. The patches were secured for a 4 h exposure period and removed with distilled water. At 24 h and 48 h after the application of PG-E, skin irritation was observed, and the extent of the evident skin allergic reaction was evaluated for each test animal. The acute primary irritation test was applied following the classic Draize test [17]. 

### 3.5. Anti-Bacterial Activities against P. acnes and S. aureus

*P*. *acnes* (BCRC10723) and *S*. *aureus* (BCRC 10781) were obtained from the Bioresource Collection and Research Center (Hsinchu, Taiwan). *P*. *acnes* was cultured in BAP with an anaerobic atmosphere using MGC AnaeroPack-Anaero and MGC AnaeroPack-Jar (Mitsubishi Gas Chemical Company). *S*. *aureus* was cultured in Bacto™ TSB (Difco). PG-E, punicalagin (**1**), punicalin (**2**), strictinin A (**3**), and granatin B (**4**) were tested against *P*. *acnes* by the paper disc-diffusion method. All experimental procedures were performed according to our previous study with slight modification [17]. Freshly grown *P*. *acnes* was diluted with Bacto™ TSB, and 10 mL of prepared bacteria (4 × 10^8^ CFU/mL) was aseptically added to 90 mL of sterilized media (TSA) at 45 °C in a water bath. The seeded agar media were immediately mixed and poured into a Petri dish. Prepared sample discs (8 mm in diameter) were added. In addition, 10 U of penicillin was used as the positive control. Plates were incubated under anaerobic conditions at 37 °C for 24 h, and inhibition zones in millimeters were measured. Each experiment was repeated in triplicate. The experimental protocol of anti-*S*. *aureus* (0.5 × 10^8^ CFU/mL) activities was the same as that described above with some modifications. The anti-bacterial activity is expressed as the diameter of the inhibition zone against the test microorganisms as follows: ratio (%) = (diameter zone of sample/diameter zone of penicillin) × 100. An inoculation loop was used to collect bacteria from the clear zone on an agar plate. The inoculation loop was enriched in TSB for 24 h. At the end of incubation, TSB was put onto the surface of nutrient agar plates and incubated at 37 °C for 24 h. The minimum bactericidal concentration (MBC) was estimated as the lowest concentration of the samples where no visible growth was observed. The minimum inhibitory concentration (MIC) was estimated as the lowest concentration of the samples where visible growth was observed. 

### 3.6. Scanning Electron Microscopy (SEM)

*P*. *acnes* and *S*. *aureus* were cultured in TSB to the exponential phase. After counting bacterial numbers, *P*. *acnes* and *S*. *aureus* were seeded in 96-well plates and incubated with samples at 37 °C for 12 h. The culture broth was centrifuged at 10,000 rpm for 10 min and washed with phosphate-buffered saline (PBS) twice. Pellets were fixed with 2.5% buffered glutaraldehyde at 4 °C for 2 h and then post-fixed in 1% buffered osmium tetroxide for 2 h. After the fixation process, bacteria were dehydrated in a graded series of ethanol concentrations (35%, 50%, 70%, 85%, 95%, and 100%, *w*/*v*), and these solvents were replaced in turn with liquid carbon dioxide by critical point drying. After mounting onto carbon stubs and vacuum sputter-coating with gold, the samples were analyzed with a Hitachi S-2400N SEM (Tokyo, Japan) under standard operating conditions. 

### 3.7. Anti-Lipase Activity

Anti-lipase activity was evaluated by a fluorometric assay. PG-E and its active components were reacted with a lipase solution (100 U/mL) and a 4-methylumbelliferyl butyrate solution (10 µM), and the reaction was terminated using sodium citrate (0.1 M) [30]. Fluorescence was detected in a fluorometric microplate reader (λex: 365 nm, λem: 445 nm). Each experiment was performed in triplicate. Results are expressed as the mean ± standard deviation (SD), and the percentage of inhibition was compared to the control.

### 3.8. Anti-Proliferative Activity against Testosterone-Treated HaCaT Cells

HaCaT cells, a human keratinocyte cell line, were cultured in DMEM with 10% FBS, 1% penicillin-streptomycin, and 1% l-glutamine at 37 °C and 5% CO_2_. HaCaT cells (2 × 10^4^ cells/well) were seeded in 96-well plates and co-treated with testosterone (10 µg/mL), and test samples were incubated for 18 h [31]. Cell proliferative activity was detected by an MTT assay.

### 3.9. Anti-Inflammatory Activity against Heat-Killed P. acnes-Treated RAW 264.7 Cells

*P*. *acnes* (4 × 10^8^ CFU/mL) was incubated at 80 °C for 30 min to prepare heat-killed *P*. *acnes* (HKP). The HKP was freeze-dried and stored at 4 °C until use. RAW 264.7 cells (BCRC-60001) were cultured in DMEM with 10% FBS, 1% penicillin-streptomycin, and 1% l-glutamine at 37 °C and 5% CO_2_. RAW 264.7 cells (8 × 10^4^ cells/well) were seeded in 96-well plates and co-treated with HKP (100 µg/mL) and test samples [32]. After 18 h, nitric oxide (NO) production was determined by mixing the cell culture medium with a Griess reagent, and the absorption was detected in an enzyme-linked immunosorbent assay (ELISA) reader at 530 nm. Prostaglandin E_2_ (PGE_2_) concentrations in cell culture media were determined with a PGE_2_ ELISA kit (Enzo Life Sciences, Farmingdale, NY, USA). Cell viability was detected by an MTT assay.

### 3.10. Anti-Inflammatory Activity against Heat-Killed P. acnes-Treated THP-1 Cells

THP-1 cells, a human monocytic cell line (BCRC-60430), were cultured in RPMI with 10% FBS, 1% penicillin-streptomycin, and 1% l-glutamine at 37 °C and 5% CO_2_. THP-1 cells (1 × 10^6^ cells/well) were seeded in 24-well plates and co-treated with HKP (100 µg/mL) and test samples for 18 h. TNF-α and IL-8 concentrations of cell culture media were determined by respective enzyme immunoassay kits.

### 3.11. Statistical Analysis

Data are presented as the mean and SD. Significance was calculated using Student’s *t*-test and a one-way analysis of variance (ANOVA) by SPSS software v.15 (SPSS, Chicago, IL, USA).

## 4. Conclusions

In this study, we used several approaches to explore the multiple therapeutic effects of PG-E against *P*. *acnes*. Our results showed that PG-E directly attenuated *P*. *acnes*-induced Wistar ear edema in vivo and inhibited the growth of acne-related bacteria, i.e., *P*. *acnes* and *S*. *aureus*, down-regulated sebum production by reducing lipase activity, attenuated the inflammatory status, and avoided keratinocyte over-proliferation in vitro. Taken together, we suggest that the 70% acetone PG-E showed multiple anti-acne capacities, including anti-bacterial, anti-lipase, anti-keratinocyte proliferation, and anti-inflammatory actions. Four hydrolysable tannins, punicalagin, punicalin, strictinin A, and granatin B, were isolated from 70% acetone PG-E and exhibited different anti-acne effects. However, punicalagin, which had the highest effect on various functions, can be employed as a quality control marker. Pomegranate has great potential to be developed as a dietary supplement and for use in skin-care products. 

## Figures and Tables

**Table 1:** The pathogenic mechanism of acne and experimental design of our platform.

Pathogenesis of Acne Vulgaris	Epithelial Cell & Keratin Accumulation	Sebum Accumulation	Bacterial Growth	Skin Inflammation
In vitro experimental designs	Testosterone-induced HaCaT cell proliferation	Anti-lipase activity	*Propionibacterium* *acnes* *Staphylococcus* *aureus*	Heat-killed *P*. *acnes*-induced RAW 264.7 Heat-killed *P*. *acnes*-induced THP-1
Effective hydrolysable tannins	Punicalagin (**1**)	Punicalin (**2**)	Strictinin A (**3**)	Granatin B (**4**)

*P. acnes*: *Propionibacterium acnes.*

**Table 2:** Anti-*P*. *acnes* and *S*. *aureus* effects of pomegranate extract (PG-E).

PG-E Samples	Diameter of Inhibition Zone (mm)
PG-E (mg/disc)	*P*. *acnes*
0.25	11.3 ± 0.6
0.5	15.1 ± 0.5
1.0	17.1 ± 1.4
PG-E (mg/disc)	*S*. *aureus*
2.5	12.6 ± 0.3
5.0	15.4 ± 0.9
10.0	15.9 ± 0.7

**Table 3:** Anti-*P*. *acnes* and *S*. *aureus* effects of pomegranate extract (PG-E).

Anti-Bacterial Activity				
MIC/MBC (µg/mL)	1	2	3	4
*P*. *acnes*	6.25/12.5	6.25/12.5	12.5/25	100/-
*S*. *aureus*	12.5/25	12.5/25	25/50	12.5/25

MIC: minimum inhibitory concentration; MBC: minimum bactericidal concentration.
